# Temperature‐Related Differences in Hair Cortisol Among Outdoor‐Housed Rhesus Macaques (*Macaca mulatta*)

**DOI:** 10.1002/ajp.70030

**Published:** 2025-04-04

**Authors:** Alexander J. Pritchard, Rosemary A. Blersch, Brenda McCowan, Jessica J. Vandeleest

**Affiliations:** ^1^ California National Primate Research Center University of California Davis Davis California USA; ^2^ Department of Population Health & Reproduction, School of Veterinary Medicine University of California Davis Davis California USA

**Keywords:** captive, environmental stress, glucocorticoids, thermoregulation, welfare

## Abstract

Temperature has a known potential to influence glucocorticoid concentrations obtained from fecal samples in nonhuman primates. Studies reliant on hair cortisol estimates obtained using samples from outdoor subjects, however, may not control for temperature. This omission is despite the general utility of hair as a sample matrix with relatively longer periods of accrual time. We examined these dynamics in rhesus macaques (*Macaca mulatta*); importantly, this population of rhesus macaques exhibits covariation between season and breeding behavior. Thus, we also examined temperature relative to contributions of social behavior associated with breeding seasons, which may coincide with climatic shifts. We utilized 1921 hair samples from 580 subjects across six large outdoor‐housed mixed‐sex groups at the California National Primate Research Center to quantify the capacity for warmer or cooler outdoor temperatures to influence hair cortisol concentrations. We found that colder maximum temperature estimates over the days preceding hair sampling were associated with elevated hair cortisol concentrations, relative to warmer periods. Temperature contributed similarly in a model with a reduced data set (1418 samples) which included breeding‐associated social behaviors. Consortship behavior was associated with hair cortisol without temperature, but was not associated with temperature included. Aggression was associated with cortisol, with or without the inclusion of temperature. Outdoor temperature is an important confound or covariate to account for statistically or via careful study design. Inclusion is especially important among research projects reliant on hair cortisol from outdoor‐housed primates and spanning multiple seasons.

## Summary

- We utilized 1921 hair samples from 580 subjects in six large outdoor‐housed groups.
- Colder maximum temperatures were associated with elevated hair cortisol, relative to warmer maximum temperatures.
- Inclusion of behaviors that increase in breeding season showed distinct associations with cortisol, when temperature was included or omitted. Outdoor temperature is a salient variable for studies reliant on hair cortisol.

## Graphical abstract

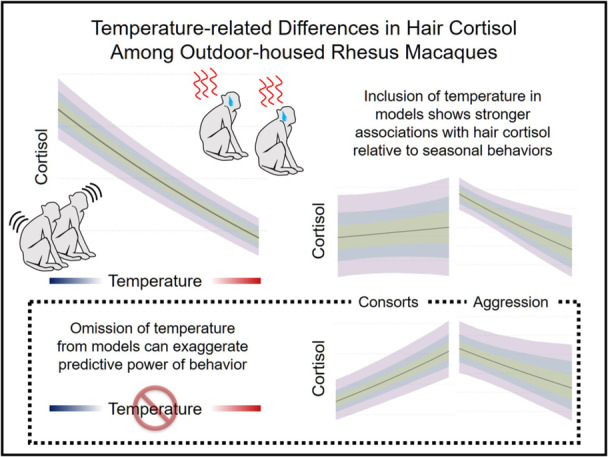

## 1 Introduction

Despite the known potential for effects of temperature on glucocorticoid concentrations, captive studies with outdoor animals underexplore temperature's effects (Takeshita et al. 2014). This oversight persists in studies utilizing hair cortisol. Long‐term studies or research using physiological samples with longer‐term accrual patterns could easily be biased by temperature effects. Indeed, hair is often selected because of its capacity to accrue cortisol concentrations over a temporally extended period relative to other biological matrices. Therefore, temperature persists as a salient nuisance or confounding variable that can statistically mask desirable effects, but which can be easily accounted for analytically. Examination of temperature as a variable of interest, however, also increases our understanding of nuanced interactions within complex systems.

Equivocal evidence has been reported for the covariation of temperature and fecal glucocorticoids (fGCs) among wild primates. Concentrations of fGC metabolites (fGCm) have shown negative (e.g., Beehner and McCann 2008), positive (e.g., Gesquiere et al. 2008; Pritchard et al. 2025), and null associations (e.g., Gesquiere et al. 2011) with seasonal variation. Thus, the association between temperature and fGCms is either not ubiquitous across wild primate populations or its effect size may be low (MacLarnon et al. 2015, and citations within). The influence of temperature on fGC concentrations in outdoor‐housed captive primates has been emphasized by a small number of studies relative to the number of studies that rely on such biomarkers. Two studies reported covariance between colder periods and heightened fGC concentrations, relative to warmer periods, among outdoor‐housed female Japanese macaques (*Macaca fuscata*) (Nelson 2020; Takeshita et al. 2014). These studies were limited in subject sample sizes (37 and 17 subjects, respectively), relied on fecal matrices, and did not include social behavior that may co‐vary with seasonal shifts (though Nelson 2020 included feeding and movement behaviors).

### 1.1 Association of Temperature With Hair Cortisol

Given the extended accrual period and temperature's known effect on cortisol in other biological matrices, we examined whether temperature was associated with hair cortisol concentrations. To do so, we elected to draw upon our own collection of hair cortisol samples to quantify and demonstrate the potential effect size of temperature on hair cortisol concentrations. In prior work using hair samples, we reported a low certitude that temperature contributed to cortisol concentrations among developing (3 mo.–3 yo.) rhesus macaques (*M. mulatta*) (Pritchard et al. 2023). Our sampling events, however, were tightly coupled with temporal and age‐related progression from a small subset of young individuals. Here we aimed to quantify the effect of temperature on hair cortisol from a larger, predominantly adult, population of outdoor‐housed rhesus macaques.

Cortisol and temperature changes could be mechanistically coupled via physiological and behavioral thermoregulation through metabolic and stress‐induced (allostatic load) pathways (de Bruijn and Romero 2018). The multidirectional and complex interactions of these mechanisms impede straightforward predictions. For example, high temperatures impede the binding of GCs to carrier proteins, yet levels of binding proteins can vary seasonally and even increase in summer (Boonstra et al. 2001; Cameron et al. 2010; Delehanty and Boonstra 2011). To add to this complexity, GCs are a metabolic hormone that exhibit an association with energetic expenditure (Markham and Gesquiere 2017; Muller and Wrangham 2004), yet high levels of physical activity can promote diurnal regulation (Moyers and Hagger 2023). As endotherms, rhesus macaques control and protect their internal temperature via behavioral and physiological pathways which can both utilize cortisol (de Bruijn and Romero 2018). Behavioral thermoregulation in primates occurs in response to both cold and hot temperatures, though the energetic budgeting of these dynamics is poorly documented, especially in naturalistic conditions (Thompson and Hermann 2024). Thus, we explore whether temperature extremes in either direction might alter cortisol concentrations as animals work to maintain their optimal internal temperature.

Generally, we hypothesized that more extreme temperatures will heighten hair cortisol in rhesus macaques with multiple predictions supported by evidence from nonhuman primates and other animals. Relatively higher temperatures will be associated with increased hair cortisol concentrations (de Bruijn and Romero 2018; Gesquiere et al. 2008; Pritchard et al. 2025; but see Gesquiere et al. 2011; Schoof et al. 2016). Alternatively, relatively cooler temperatures will be associated with increased hair cortisol concentrations (Beehner and McCann 2008; Cunha et al. 2007; de Bruijn and Romero 2018; Nelson 2020; Takeshita et al. 2014; Weingrill et al. 2004). Finally, both of these predictions could be supported whereby extremes in either direction increase cortisol relative to intermediate temperatures. This occurrence could arise if both extremes activate thermoregulatory action to maintain homeostasis and would result in a nonlinear u‐shaped relationship between temperature and cortisol.

### 1.2 Association of Temperature and Behavior With Hair Cortisol

Seasonal patterns of behavior might show variation in cortisol that could be attributable to, either, climatic seasonal changes or the behaviors themselves. That is, though temperature might operate as an independent variable of interest, we acknowledge that there are socio‐behavioral and demographic effects that co‐occur with seasonal fluctuations which have not been fully explored. For instance, peak breeding seasons typically occur in cooler months (i.e., September–November, respectively) among rhesus macaques at the California National Primate Research Center (CNPRC). Thus, we aimed to analytically disentangle these complex dynamics by including breeding‐associated behaviors of interest. This aim is important because studies have shown equivocal effects of breeding season on GC concentrations with analyses of broad seasonal categories as proxies for periods of variation in reproductive behavior (McFarland et al. 2013). The use of mating‐relevant behaviors, however, has revealed differences in fGCm concentrations for behaviors relevant during the breeding season. That is, individuals with higher rates of mounting or copulatory success have been shown to have lower concentrations of fGCm or circulating plasma GC, relative to individuals with lower rates (McFarland et al. 2013; Sapolsky 1982). On a broader timescale, however, high‐ranking males exhibit higher fGCms during periods of high copulations relative to periods with lower copulations (Milich et al. 2018). Both Milich et al. (2018) and McFarland et al. (2013), however, do not incorporate explicit measures of environmental variation (e.g., temperature or rainfall). This caveat is important to emphasize given that many populations of rhesus macaques exhibit seasonal breeding that are partially entangled with climatic cues (i.e., *zeitgebers*) (Oates‐O'Brien et al. 2010; Rawlins and Kessler 1985; Small 1990; Stavisky et al. 2018; but see Hernández‐Pacheco et al. 2016).

A composite understanding of changes in environmental cues and associated changes in breeding‐associated behaviors with regard to GC concentrations has been poorly resolved. Thus, we incorporated behaviors associated with breeding season: aggression (Stavisky et al. 2018; Theil et al. 2017; Wilson and Boelkins 1970) and consortships (Small 1990), the latter of which involves near constant social contact between two individuals (see Methods). We aimed to disentangle the social dynamics of breeding season and climatic dynamics. Both components have been shown to independently influence GC concentrations, but they temporally co‐vary. Prior research indicates that GCs should (a) increase during the breeding season (Milich et al. 2018), when consortships and aggression rates are high; and (b) increase disproportionately higher among low‐ranked individuals (McFarland et al. 2013; Sapolsky 1982). As the breeding season coincides with shifts toward colder temperature, we sought to determine whether greater variation in our datasets was explained by behavioral or climatic variables. We sought to assess the relative contributions of breeding‐associated behaviors as compared to temperature, and assess whether the omission of temperature might markedly alter interpretations.

## 2 Methods

### 2.1 Study Population

We studied 580 subjects (403 females; 177 males) from six socially housed groups (Table 1). Subjects were outdoor‐housed at the California National Primate Research Center (Davis, CA, USA) in half‐acre enclosures among large mixed‐sex groups. These groups had continuous full‐group contact in naturalistic matrilineal formations approximating the sex ratio of wild macaques (Pritchard et al. 2024). Each enclosure housed a unique social group. Fully adult animals were housed in these enclosures throughout the study unless relocated by animal management. Enclosures had grass or gravel substrates, A‐frame perches, enrichment objects (play structures, barrels), front feeders, and ad libitum access to feed as well as water. Our work appropriately adhered to the Principles for the Ethical Treatment of Non‐Human Primates, set by the American Society of Primatologists, and included approval by the University of California, Davis’, Institutional Animal Care and Use Committee.

**Table 1:** Subject (*N*) and sample (*n*) counts divided by social group and sex, with descriptive statistics (mean ± SD) for subject age and the number of samples per subject.

		Groups					
Sex	Descriptive	A	B	C	D	E	F
Females	*N* =	75	105	57	47	52	67
	Age (*M* ± SD)	8.85 ± 5.73	7.69 ± 5.36	9.72 ± 5.71	5.74 ± 2.38	6.40 ± 2.59	6.57 ± 2.57
	*n* =	192	537	164	135	201	162
	*n*/*N* (*M* ± SD)	2.56 ± 0.83	5.11 ± 2.82	2.88 ± 0.43	2.87 ± 0.40	3.87 ± 0.49	2.42 ± 0.63
Males	*N* =	27	50	24	23	20	33
	Age (*M* ± SD)	5.52 ± 3.47	4.49 ± 3.75	4.71 ± 3.62	4.87 ± 2.60	5.40 ± 2.80	4.61 ± 2.00
	*n* =	77	164	72	54	75	88
	*n*/*N* (*M* ± SD)	2.85 ± 0.53	3.28 ± 1.55	3.00 ± 0.00	2.35 ± 0.78	3.75 ± 0.64	2.67 ± 0.65
Total	N =	102	155	81	70	72	100
	*n* =	269	701	236	189	276	250

### 2.2 Biological Samples

We utilized 1921 hair cortisol samples assayed across 8 years of intermittent sampling from the 580 subjects (Table 1). These samples were collected during prior projects involving behavioral or sociodemographic manipulations. Samples were batch collected from animal subjects across 27 discrete collection events. We resampled the same groups typically 5‐to‐24 weeks apart—we only sampled subjects ≥ 3 years of age, except for samples from group “B” collected after 2015. Groups were not uniformly sampled across years: thus, per group, we had an average of 320.17 samples (minimum = 189, maximum = 701), with a per subject average of 3.31 samples (±1.67 SD). The median age of our subjects was 5 years of age (±6 IQR), and 82% of our samples were from subjects ≤ 10 years of age.

Hair cortisol was extracted from the whole hair strand and was assayed as has been previously validated using enzyme immunoassays (Salimetrics, Carlsbad, CA, USA) (Pritchard et al. 2023; Vandeleest et al. 2019). Assays passed interassay and intrassay coefficients of variation thresholds of 15%. We utilized pooled adult samples as quality controls within and across plates. Pooled samples had a mean intraassay CV of 2.28% ± 2.14SD, after exclusion of two quality controls on distinct plates with high CVs (59.53% and 13.01%), with clear aberrancies in estimated cortisol in one or two of the wells—each plate had a remaining quality control that was as expected. Interassay coefficients of variation were 13.92% based on adult pooled samples replicated across plates. Samples with an intraassay CV ≥ 15% were re‐run. Retained samples had a mean intraassay CV of 2.40% ± 1.96SD.

### 2.3 Behavioral Sampling

Behavioral data were collected for six of the cages during the same sampling periods as their respective biological samples (2013–2016). Our behavioral data protocols involved collecting data 4 days a week for 6 h a day, as previously described (Balasubramaniam et al. 2019; Balasubramaniam et al. 2016; Pritchard et al. 2024).

Conflict data were collected using an event sampling design (Altmann 1974). We retained data where identities were known for both dyadic partners. To avoid “chains“ of complex polyadic bouts, we limited our conflict analyses to ‘directed’ dyadic aggression or ‘redirected’ aggression by excluding triadic or polyadic interventions and policing. Behaviors were collected into bins based on severity of aggression. Individual rates of outgoing high‐ or low‐level conflict were moderately correlated (*r* = 0.52), thus we pooled aggression across these bins. We extracted three measures of aggression data: individual rates of conflict given, individual rates of conflict received, and total conflict rates per group. These rates were calculated per observation period and normalized by observation days.

Consort information was collected during scan samples for affiliative data (Altmann 1974). Consortships were not strictly defined to exhibiting sexual behavior (i.e., mounting and copulations were not required) and were scored among different‐ and same‐sex pairs. Consortships were defined by near‐constant social contact for over 30 min during social observations, typically with individuals walking shoulder‐to‐shoulder or following closely (within 1 m) and engaging in frequent affiliation. Dyads were only recorded as consorting once‐per‐day. To avoid pseudo‐replication of consorting pairs across sample days, we tallied the number of unique consorting pairs per study period. As the number of days of observation varied across study periods, we normalized the number of unique consorting pairs by observation days within each period. Rates of consortships were moderately correlated with temperature estimates (*r* = −0.46). The observed incidence of consortships was seven times greater when temperatures were ≤ 25°C than when they were > 25°C.

### 2.4 Temperature and Climatic Metrics

Temperature readings were recorded at the University of California Davis’ Russell Ranch weather station (UCD/NOAA Climate Station: USC00042294 [Atmospheric Science Program, Land Air and Water Resources, University of California Davis 2023; Menne et al. 2012]). We utilized three temporal windows for temperature (2, 7, or 14 days before sample collection). We selected 14 days as a maximum window given that an injection of radio‐labeled cortisol was fully incorporated into rhesus macaque hair by this time (Kapoor et al. 2018). We note, however, a lack of formal consensus on the temporal biological accumulation and retention: several lines of evidence indicate that the time‐span for uptake may be shorter than 2 weeks and that even acute ongoing stressors can alter hair cortisol concentrations (Cattet et al. 2014; Colding‐Jørgensen et al. 2023; Kalliokoski et al. 2019; Kapoor et al. 2018; Sharpley et al. 2010, 2009). Thus, we selected 2‐ and 7‐day windows to accommodate the rationale that hair cortisol may represent both short‐term and longer‐term responses. We selected 2 days as a lower‐bound. This is because hair cortisol has been shown to be most correlated to fGC measures, relative to other sampling matrices (Kalliokoski et al. 2019). Fecal GCs are linked to gut‐transit time which approximate 48 h of accumulation in other monkey species (Wasser et al. 2000). As daily minimum temperatures occurred in the morning before sample collection, minimum temperature included day of sample collection. Maximum daily temperatures, however, would peak after sample collection and were calculated from the day prior.

For both maximum and minimum temperature, we aggregated: (1) the mean average temperature given longer periods of extreme temperature might be more influential on hair cortisol; (2) the warmest (maximum) or coolest (minimum) temperature given extreme values might have a disproportionate influence; or, (3) a decaying accrual whereby older temperatures were penalized given temperatures closer to sampling might be more influential. Penalization was computed via linearly weighted normalization whereby the oldest sample was multiplied by 0.1, the newest sample by 1.0, and intervening samples by an evenly spaced linear sequence between 0.1 and 1.0 equivalent in length to the intervening days between oldest and newest dates. The resulting values were summed, and then divided by the sum of the interval sequence. We used these three metrics for each of the three time windows, for both the maximum and minimum daily temperatures totaling 18 alternative temperature estimates (3 × 3 × 2).

Across temporal windows, temperature estimates were highly correlated (*r* ≥ 0.91). Within aggregating approaches, minimum and maximum temperature estimates were highly correlated (*r* between 0.80 and 0.92). Similarly, temperature measures were highly correlated within minimum and maximum temperature aggregates, irrespective of the temporal window (*r* ≥ 0.90 and 0.92 within minimum and maximum measures, respectively). Within our data set, the coldest minimum was −2.96°C and the warmest maximum was 41.79°C. The minimum and maximum 2‐day averages were 8.12°C and 25.30°C, respectively. Mean maximum and minimum temperature estimates for our sampling events were fairly representative of general annual temperature fluctuations within the region (Supporting Information S1: Figure 1).

### 2.5 Model Structures

#### 2.5.1 Association of Temperature With Hair Cortisol

We ran Bayesian Regression Models (“brms”) using Stan (Bürkner 2017) in R and specified weakly informative priors throughout. We constructed initial models with all six groups. Inclusion of random slopes for temperature across the groups (1 + Temperature|Group); however, revealed that Group “B“ had a markedly shallower slope than the other five groups, which shared a similar slope (Supporting Information S1: Figure 2). Five groups had been sampled with three sampling events (except for “E,” which had been sampled four times) each 5–8 weeks apart across ~3 months. Group “B,” however, had been sampled for a longer duration (2014–2020). In 2014, Group “B“ was sampled similarly to the other groups. From 2016 to 2020, Group “B“ had a lower‐density sampling period with each event approximately 24 weeks apart. Only mothers and infants were sampled during this latter period. Thus, we made the decision to partition the initial models due to these sampling and analytical differences. We built two models to assess the effect size of temperature without including behavior: one for five groups (“A,” “C,” “D,” “E,” and “F“) and one for group “B.”

After sample pre‐screening for outliers (Supporting Information S1: 1), we retained 1220 samples from 425 subjects in the five‐group model and 695 samples from 155 subjects in the “B“ group model. Model selection procedures using expected log predictive densities (*loo_compare* in the brms package; Bürkner 2017) revealed that temperature aggregates performed similarly. Generally, models with the hottest 2‐day maximum temperature estimates were favorable for the five‐group model (Supporting Information S1: 2). We used the same temperature estimate for the “B“ group model.

Our final five‐group model retained temperature interacting with age and sex as fixed effects with group and subject ID as random effects. Our “B“ group model retained temperature, age, and sex as fixed effects with subject ID as a random effect. Both models used 6500 iterations, across 4 chains, with a warmup of 1500 and a thin of 2. Model fit was acceptable based on $\hat{R}$ values (1.00), effective sample size (ESS) values, as well as visual inspection of *pp_check* density, loo‐pit, and *pairs* plots (Supporting Information S1: 3 and Figures 3 and 4).

### 2.6 Association of Temperature and Behavior With Hair Cortisol

We built several models with consortship and aggression rates at the group and individual levels to assess the relative contributions of temperature compared to breeding‐associated behavior. These data were matched by 1418 hair cortisol samples from 521 subjects, across six groups. We included the data from Group “B“ as these data were sampled in 2014 when a similar sampling structure was used across all groups. We performed model selection in parallel: with and without temperature; for simplicity, we did not include interaction terms. During model selection, we used 2000 iterations, across two chains, with a warmup of 500 and no thin. The inclusion of temperature uniformly improved model performance (Supporting Information S1: 4 and Table 1) such that, relative to the top performing temperature‐behavior model, the behavior‐only model had markedly worse predictive performance (elpd_diff = −147.4 ± 18.3SE). In the temperature‐behavior models, all but the top‐performing model was similar, but the second‐best model did not perform similarly to the top model (elpd_diff −10.0 ± 3.5SE). The top‐ranked model included aggression given, consorts, sex, age, and temperature as fixed effects with group and subject ID as random effects.

For the behavior‐only models, two models performed similarly. We proceeded with the second‐ranked model because the top‐performing model exhibited evidence of collinearity between aggression given and received. This model outperformed the four alternatives, with the third‐ranked model having an elpd_diff of −24.7 ± 5.5SE, relative to the second‐ranked model. The second‐ranked model was identical to the temperature‐behavior model albeit without temperature. For full models, we ran 6500 iterations, across 4 chains, with a warmup of 1500 and a thin of 2. Model fit of reported models was acceptable, based on $\hat{R}$ values, ESS values, as well as visual inspection of *pp_check* density, loo‐pit, and *pairs* plots (Supporting Information S1: Figures 5 and 6).

## 3 Results

### 3.1 Association of Temperature With Hair Cortisol

Among the five groups, cooler maximum temperatures over the 2 days before sample collection were credibly associated with elevated hair cortisol (pg/mg) relative to samples from warmer periods (estimate = −0.24; 95% CIs = −0.27 to −0.21) (Table 2). We found relatively smaller effects of age as well as a temperature: age interaction. Older individuals had higher cortisol relative to younger individuals (estimate = 0.08; CIs = 0.03–0.12). Temperature and age had a credible interaction whereby older individuals had a reduced temperature responsivity in their cortisol concentrations (i.e., reduced slope), relative to younger individuals (estimate = 0.09; CIs = 0.04–0.15). Hair cortisol did not meaningfully differ between the sexes (male estimate = −0.01; CIs = −0.06 to 0.04, reference group = female). Effect sizes (using *bayes_R2*) indicated robust predictive power of the full model (conditional *R* ^2^ = 0.47 ± 0.02) with moderate contributions of the fixed effects to the variance explained by the model (marginal *R* ^2^ = 0.16 ± 0.02).

**Table 2:** Model output for the five‐group model (A) and the “B” group model (B). Both models were lognormal, with identity links. The five‐group model had 1186 observations, while “B“ group had 695.

(A)	Estimate	Est. error	Lower 95% CI	Upper 95% CI	$\hat{R}$	Bulk_ESS	Tail_ESS
Group‐level effects							
~Subject_ID, *N* = 425, SD (Intercept)	0.17	0.01	0.15	0.18	1	4794	5803
~Group, *N* = 5, SD (Intercept)	0.08	0.05	0.03	0.23	1	2328	1503
Population‐level effects							
Intercept	3.96	0.04	3.87	4.05	1	2617	2135
Temperature_Max	−0.24	0.01	−0.27	−0.21	1	8007	8861
Age	0.08	0.02	0.03	0.12	1	5555	6637
Sex (male)	−0.01	0.02	−0.06	0.04	1	5191	7007
Temperature_Max: Age	0.09	0.03	0.04	0.15	1	7922	8676
Family‐specific parameters							
Sigma	0.20	0.01	0.19	0.21	1	6181	8633

Bayes_*R* ^2^ estimates							
	Estimate	Est. error	Quartile 2.5	Quartile 97.5			
Conditional *R* ^2^	0.47	0.02	0.42	0.50			
Marginal *R* ^2^	0.16	0.02	0.12	0.19			

**(B)**	**Estimate**	**Est. error**	**Lower 95% CI**	**Upper 95% CI**	$\hat{R}$	**Bulk_ESS**	**Tail_ESS**
Group‐level effects							
~Subject_ID, *N* = 155, SD (Intercept)	0.15	0.01	0.12	0.18	1	5304	7611
Population‐level effects							
Intercept	4.28	0.02	4.25	4.32	1	8089	8726
Temperature_Max	−0.05	0.02	−0.09	−0.02	1	7012	7743
Age	−0.14	0.03	−0.20	−0.08	1	7426	8787
Sex (male)	−0.05	0.03	−0.12	0.02	1	8882	9053
Family‐specific parameters							
Sigma	0.20	0.01	0.19	0.22	1	6953	7,954

Bayes_*R* ^2^ estimates							
	**Estimate**	**Est. error**	**Quartile 2.5**	**Quartile 97.5**			
Conditional *R* ^2^	0.35	0.03	0.29	0.41			
Marginal *R* ^2^	0.06	0.02	0.03	0.11			

Similarly for “B“ group, cooler maximum temperatures were credibly associated with elevated hair cortisol (pg/mg) relative to samples from warmer periods (estimate = −0.05; CIs = −0.09 to −0.02) (Table 2) although this estimate was smaller than that from the five‐group model. Age was also associated with differences in hair cortisol, but in the opposite direction as the five‐group model: younger individuals had higher cortisol (estimate = −0.14; CIs = −0.20 to −0.08). Males did not credibly differ from females as a reference group (estimate = −0.05; CIs = −0.12 to 0.02). Effect sizes (using *bayes_R2*) indicated robust predictive power of the full model (conditional R^2^ = 0.35 ± 0.03) though the fixed effects contributed less to explained variance (marginal R^2^ = 0.06 ± 0.02).

### 3.2 Association of Temperature and Behavior With Hair Cortisol

Models including temperature and behavior retained the aforementioned associations between temperature and hair cortisol (pg/mg) with a similar estimate (−0.23; 95% CIs = −0.26 to −0.20) (Table 3). Aggression given was also associated with hair cortisol such that higher rates of aggression were associated with lower concentrations of cortisol, relative to lower aggression (estimate = −0.05; CIs = −0.08 to −0.02). Though consorts were included through model selection, there was no credible association between the rate of unique consorts and hair cortisol concentrations (estimate = 0.01; CIs = −0.03 to 0.05). Hair cortisol did not credibly differ by age (estimate = 0.03; CIs = −0.01 to 0.07) or sex (male estimate = −0.01; CIs = −0.05 to 0.03, reference group = female). Effect sizes were indicative of robust predictive power for the full model (conditional *R* ^2^ = 0.48 ± 0.02) with moderate contributions of the fixed effects to the variance explained by the model (marginal *R* ^2^ = 0.15 ± 0.02).

**Table 3:** Model output for the temperature‐behavior model (A) and the behavior‐only model (B). Both models were lognormal, with identity links, and with 1418 observations.

(A)	Estimate	Est. error	Lower 95% CI	Upper 95% CI	$\hat{R}$	Bulk_ESS	Tail_ESS
Group‐level effects							
~Subject_ID, *N* = 521, SD (Intercept)	0.17	0.01	0.15	0.18	1	5666	7754
~Group, *N* = 6, SD (Intercept)	0.07	0.04	0.03	0.17	1	4146	5832
Population‐level effects:							
Intercept	4.04	0.03	3.97	4.11	1	4538	5779
Aggression (Given by ID)	−0.05	0.02	−0.08	−0.02	1	8624	9568
Consorts	0.01	0.02	−0.03	0.05	1	9377	8525
Sex (male)	−0.01	0.02	−0.05	0.03	1	6591	7742
Age	0.03	0.02	−0.01	0.07	1	6370	8216
Temperature_Max	−0.23	0.01	−0.26	−0.20	1	8577	8747
Family‐specific parameters							
Sigma	0.20	0.00	0.19	0.21	1	7113	8027

Bayes_*R* ^2^ estimates							
	Estimate	Est. error	Quartile 2.5	Quartile 97.5			
Conditional *R* ^2^	0.48	0.02	0.44	0.52			
Marginal *R* ^2^	0.15	0.02	0.11	0.18			

(B)	Estimate	Est. error	Lower 95% CI	Upper 95% CI	$\hat{R}$	Bulk_ESS	Tail_ESS
Group‐level effects							
~Subject_ID, *N* = 521, SD (Intercept)	0.16	0.01	0.14	0.18	1	4840	7387
~Group, *N* = 6, SD (Intercept)	0.13	0.06	0.06	0.31	1	2433	757
Population‐level effects							
Intercept	4.04	0.06	3.92	4.16	1	2069	738
Aggression (given by ID)	−0.03	0.02	−0.07	−0.00	1	7135	8300
Consorts	0.15	0.02	0.11	0.19	1	7459	7613
Sex (male)	−0.01	0.02	−0.05	0.04	1	8162	8695
Age	0.04	0.02	0.00	0.08	1	7744	8651
Family‐specific parameters							
Sigma	0.22	0.01	0.21	0.23	1	6111	8151

Bayes_*R* ^2^ estimates							
	Estimate	Est. error	Quartile 2.5	Quartile 97.5			
Conditional *R* ^2^	0.36	0.02	0.32	0.41			
Marginal *R* ^2^	0.06	0.02	0.03	0.10			

The behavior‐only models provided very weak to negligible evidence for an association for aggression given. That is, the estimate was small and upper credible interval approached zero to within two decimal places. Even so, the directional relationship was similar to the temperature‐behavior model. Relative to lower rates, higher rates of aggression were very weakly associated with lower cortisol (estimate = −0.03; CIs = −0.07 to −0.00) (Table 3). Unlike the temperature‐behavior model, consorts were associated with cortisol: a greater number of dyads in consortships were associated with increased concentrations of cortisol, relative to fewer consortships (0.15; CIs = 0.11–0.19). Hair cortisol did not meaningfully differ by age (estimate = 0.04; CIs = 0.00 [approached zero to within four decimal places] to 0.08) or sex (male estimate = −0.01; CIs = −0.05 to 0.04, reference group = female). Effect sizes were indicative of robust predictive power of the full model (conditional *R* ^2^ = 0.36 ± 0.02) with small contributions of the fixed effects to the variance explained by the model (marginal *R* ^2^ = 0.06 ± 0.02).

## 4 Discussion

In this paper, we have demonstrated how incorporating temperature can influence relevant analyses. Our findings highlight the association between temperature and hair cortisol levels, though explicit testing for differences between time windows of aggregation was not feasible due to model similarities. The relevance of this effect to future work is contingent upon the underlying question and what is being tested. We advocate for accounting for outdoor temperature when considering relationships between key variables and hair cortisol concentration, either through inclusion as a covariate or through careful study design.

### 4.1 Association of Temperature With Hair Cortisol

We found an association between elevated hair cortisol and cooler maximum temperatures in the 2 days prior sample collection relative to samples from warmer periods. These results parallel those of fGC concentrations in captive outdoor‐housed macaques (Nelson 2020; Takeshita et al. 2014). We extend these associations into hair as a biological sampling matrix. We emphasize that naturalistic conditions remain important components of consideration in an outdoor captive system. Studies that utilize hair cortisol from outdoor‐housed captive primates should account for temperature variation pre hoc through careful study design that does not conflate climatic fluctuations with variables of interest, or via post hoc analytics.

Long‐term responses to climatic shifts show variation across study species and temperature gradients (de Bruijn and Romero 2018). Using studies that primarily relied on blood cortisol, de Bruijn and Romero (2018) emphasized that acute perturbations elicit stronger glucocorticoid responses relative to cooler or hotter temperature exposure over longer durations. Even so, we provide evidence from a hair matrix that long‐term changes (i.e., temperature shifts on the order of weeks and months) are important for modeling cortisol concentrations. The strength of this association was not uniform across groups, with a reduced estimate in one of the groups (“B”). Group “B“ had a higher number of repeated sampling events across individuals over a longer duration. Furthermore, this group had higher sampling rates of younger individuals, important given that infants exhibit heightened cortisol (Pritchard et al. 2023) and that age showed an interaction with temperature across cages. Though we would not expect demographic properties to directly alter individual responses to temperature, we emphasize that group differences (i.e., composition, enclosure location, density, sex ratio) might be expected to alter hair cortisol concentrations. We implicitly incorporated such variation as a random effect in several of our models, but did not explicitly include these variables as fixed effects.

### 4.2 Association of Temperature and Behavior With Hair Cortisol

The addition of breeding‐associated behavior alongside temperature did not alter temperature's inclusion as a variable of interest during model selection or the magnitude of its estimate. Our model selection indicated that models including temperature were uniformly preferable compared to models without temperature. If we included temperature, individual rates of aggression given were negatively associated with hair cortisol. Higher ranking individuals exhibit greater rates of aggression given (mean *r* across groups = −0.71 between aggression given and ordinal rank [with 1 being the highest]). These results are similar to those reported at the individual level where higher ranked individuals exhibit lower concentrations of cortisol (McFarland et al. 2013; Sapolsky 1982; Zhang et al. 2018; but see Dettmer et al. 2014; Vandeleest et al. 2020).

If we excluded temperature, unique consorting dyads per observation period were associated with hair cortisol concentrations. On the surface, these associations align with prior studies that incorporated breeding‐associated behavior (McFarland et al. 2013; Milich et al. 2018; Sapolsky 1982). Consortships are expected to covary with temperature as a proxy for climatic variation (Oates‐O'Brien et al. 2010; Rawlins and Kessler 1985; Small 1990). Indeed, we found correlations between temperature and consorts, but weak to no correlation between temperature and group rates of aggression (*r* = −0.12), as well as aggression given at the individual level (*r* = −0.05). Given the stronger performance of temperature during model selection and stronger performance of temperature in the same model, it would be more parsimonious to infer that consorts are explaining variation that is subsumed by or better explained by biological responses to temperature itself.

### 4.3 Limitations

While it is tempting to emphasize our window of temperature aggregation (2 days) and our use of peak maximum temperatures during this temporal window as informative, to do so would underplay the equivocal role of these decisions in selecting our final model. That is to say, many of our models were equivalent irrespective of these assumptions. Long‐term associative studies would require careful experimental manipulations to disentangle how temperature impacts the accrual, retention, and degradation of hair cortisol. Such work should include whether acute perturbations are more impactful than overall chronic extremes.

We acknowledge that there are temporal markers that can be used as proxies for climatic variation. For instance, date or relative day of sampling could be included as a fixed or random effect. We might assume that month could account for climatic associations though such proxies also covary strongly with behavioral shifts. If our intention were simply to control for the possibility of temperature influencing GCs, then such an approach might be sufficient. If our intention, however, were to rule out the influence of climatic variables then their explicit incorporation is necessary. When relying on GCs, we advocate for the examination and incorporation of explicit climatic variables in areas with unregulated housing conditions or free‐ranging subjects. This recommendation is reasonable given that climatic variables are globally available from weather stations.

## 5 Conclusion

Our sampling events were relatively representative of the temperature variation observed in the region during the study period. The hottest and coolest temperatures we observed were well within the extreme temperature bounds that wild rhesus macaques inhabit (Lindburg 1971; Tian et al. 2013). Even so, CNPRC implements protective measures to enhance animal welfare during periods of climatic extremes via heating units and cooling sprinklers. Such facility measures could mitigate the effects of extreme temperatures. The mechanistic links between physiological biomarkers and behavioral or physiological thermoregulation would be important to parse out in a climatic context, relative to peak heat or cold stress. Such investigations are interesting in the context of our findings given the range of Primates, as an order, is more cold constrained relative to other mammalian taxa (Fleagle and Gilbert 2006).

## Author Contributions

**Alexander J. Pritchard:** conceptualization (lead), data curation (lead), formal analysis (lead), methodology (lead), visualization (lead), writing – original draft (lead). **Rosemary A. Blersch:** formal analysis (supporting), methodology (supporting), writing – review and editing (equal). **Brenda McCowan:** funding acquisition (lead), project administration (lead), resources (lead), supervision (equal), writing – review and editing (equal). **Jessica J. Vandeleest:** methodology (supporting), project administration (supporting), resources (supporting), supervision (equal), writing – review and editing (equal).

## Supporting information

Supporting information.

## Data Availability

Data and files to replicate analyses are available in the Dryad data repository: https://doi.org/10.5061/dryad.j3tx95xp2.
