# Supplementary material for: Temperature‐Related Differences in Hair Cortisol Among Outdoor‐Housed Rhesus Macaques (Macaca mulatta)
Source: Am J Primatol. 2025 Apr 4;87(4):e70030. doi: 10.1002/ajp.70030 (PMC11970219; doi:10.1002/ajp.70030)
Supplement: Supplementary file 1 — Supporting information. [file AJP-87-e70030-s001.pdf]

Supplementary Materials

Temperature-related Differences in Hair Cortisol Among Outdoor-housed Rhesus Macaques (*Macaca mulatta*)

Alexander J. Pritchard[1,2]*, Rosemary A. Blersch[1,2], Brenda McCowan[1,2], Jessica J. Vandeleest[1,2]

[1] California National Primate Research Center, University of California Davis, Davis, CA, USA

[2] Department of Population Health & Reproduction, School of Veterinary Medicine, University of California Davis, Davis, CA, USA

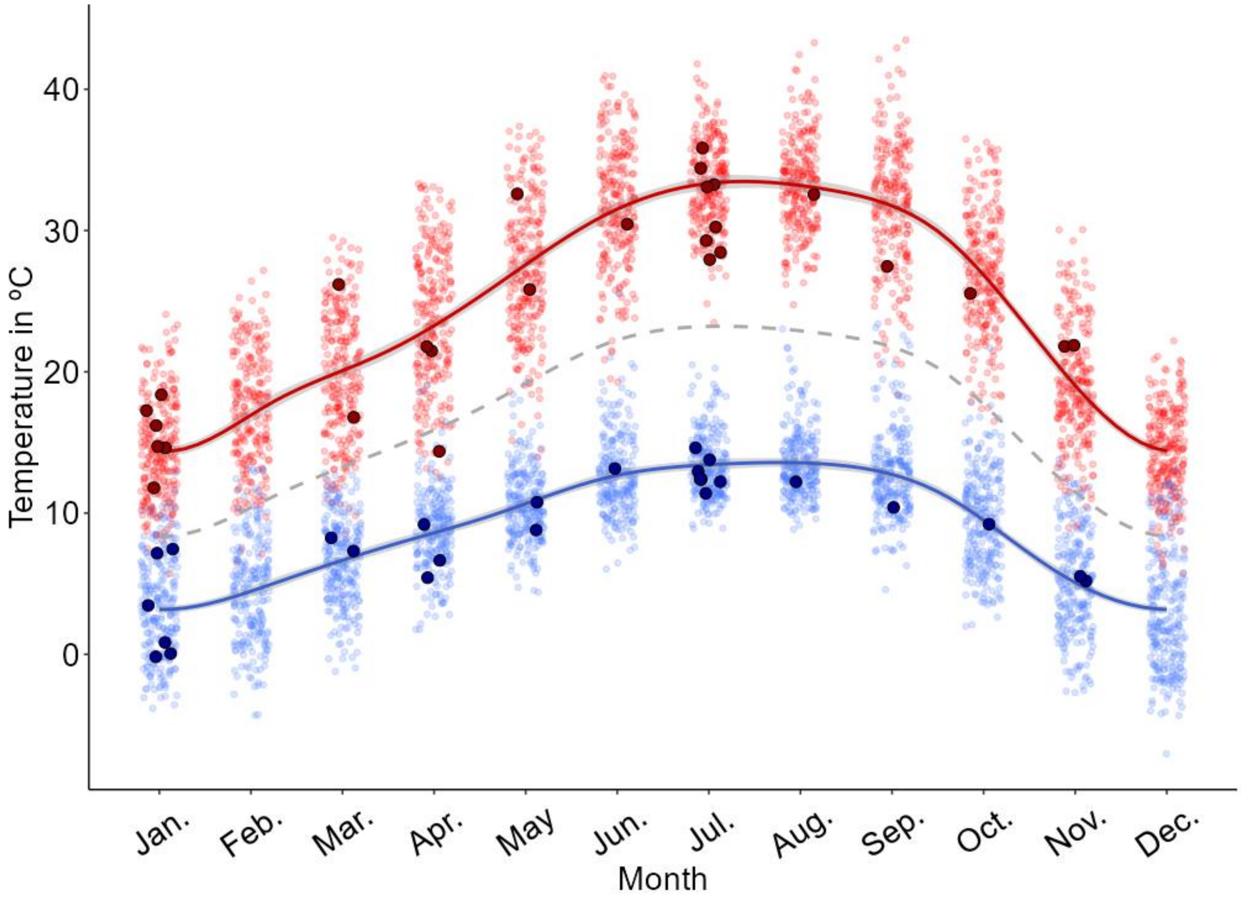

Supplementary Figure 1. Temperature estimates (y-axis) pooled by calendar month (x-axis), aggregated by daily minimum (blue) and maximum (red). Lighter background points are drawn from daily readings from 2013-2020; smooth lines are estimated using generalized-additive model cubic cyclic splines for daily maximum (red), minimum (blue), and daily mean (gray-dashed). Larger darker points visualize mean daily maximum and minimum estimates aggregated over the 7-days prior to sampling events. Sampling events were plotted based on the month of sample collection, though the period of aggregation may extend into the prior month (e.g., two of the April sampling events were on April 2).

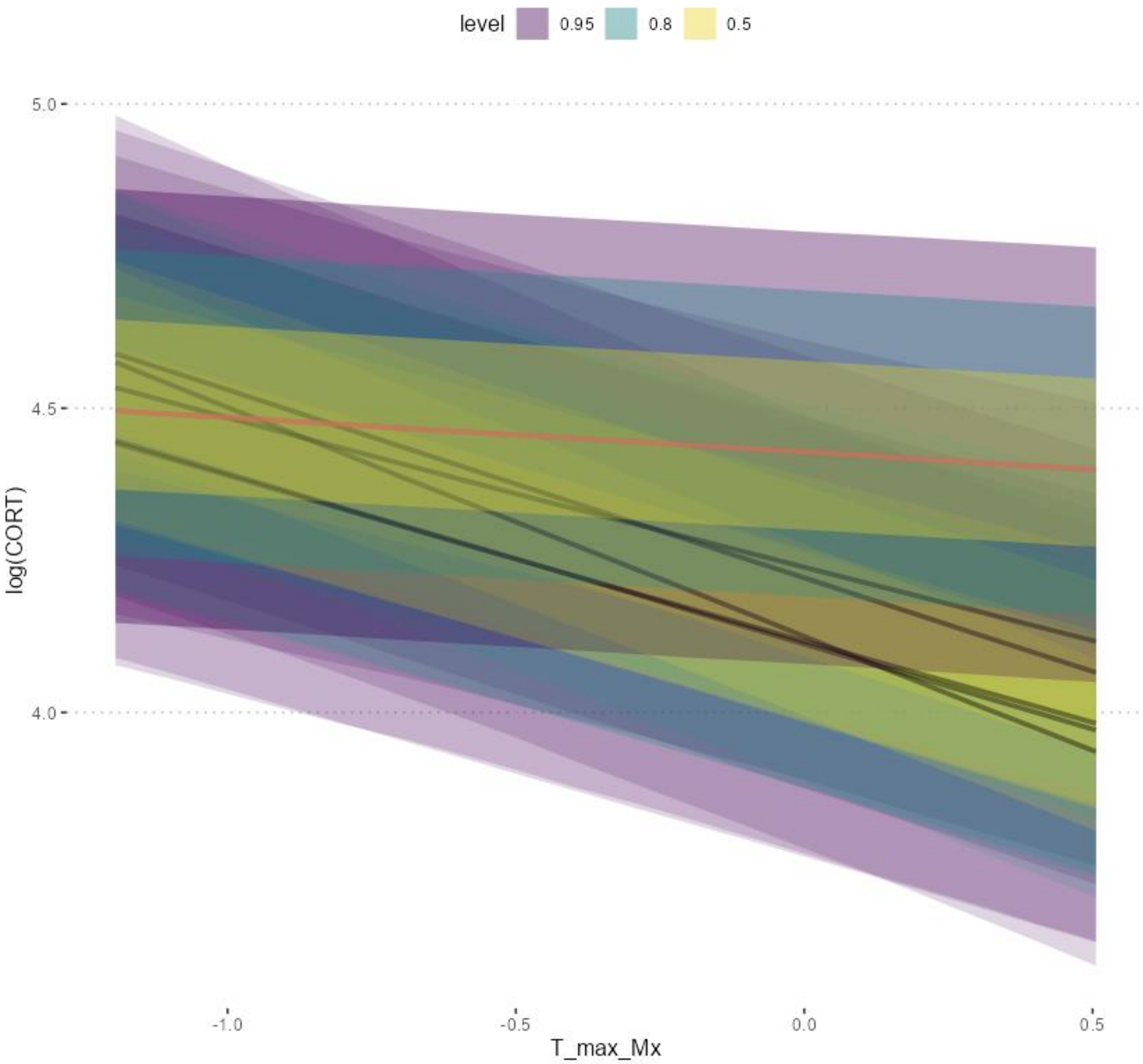

Supplementary Figure 2. Preliminary model screening with random slopes revealed strong differences in ‘B’ group relationship between temperature and hair cortisol. Random slopes obtained from posterior predictions are plotted here, with ‘B’ group’s median emphasized with a red line, compared to the median of the other groups (black lines).

S1. *Sample Pre-screening*

For the five group model, we excluded 28 samples as they were from subjects 2 years of age, as the other samples were from subjects 3 and above. Initial five group models identified 6 samples with extreme cortisol concentrations (>3*sd* from the mean) that we excluded from subsequent models: we retained 1220 samples. For the 'B' group model, we *a priori* excluded samples from subjects < 1 year of age due to previous work showing heightened cortisol among infants (e.g., Pritchard et al., 2023); those data are represented in a subset of this study's group 'B' samples (26% of these samples that are from infant and juvenile subjects). The 'B' group model had 6 samples with extreme cortisol concentrations that were excluded: we retained 695 samples. Within each dataset, temperature and age were mean centered and scaled by two standard deviations (Gelman, 2008).

S2. *Model Selection*

To select our final five group model, we first constructed an intercept-only model, confirming the suitability of a lognormal family. Subject ID and group as random effects improved fit, relative to the intercept-only model (elpd_diff = -118 ±15.8*se*). We compiled 18 models of identical structure, albeit with substitution of the 18 temperature metrics: Hair_Cortisol ~ *Temperature* + Age + Sex + (1|Group) + (1|Subject_ID). Model comparisons (*loo_compare*) suggested models with the hottest maximum 2-day aggregate outperformed the random effect model (elpd_diff = -168.7 ±18.8*se*). The decaying maximum 7-day aggregate performed similarly to the 2-day maximum (elpd_diff = -1.9 ±6.1*se*), albeit with greater uncertainty of *se* differences. Models using maximum temperature generally outperformed minimum temperature models with 8 of the 9 maximum temperatures out-ranking the minimum temperature models based on elpd_diff performance. We proceeded with the 2-day hottest maximum temperature. Models with group as a random effect outperformed group as a fixed effect (elpd_diff = -3.9 ±3.3*se*). The inclusion of interaction terms did not markedly improve fit, but supported an age*temperature interaction over a sex*temperature, or a three-way sex, age, and temperature (relative to the elpd for age*temperature, elpd_diff for sex*temperature = -0.6 ±3.6*se*; elpd_diff for age : temperature interaction with a sex : temperature interaction = -2.6 ±1.7*se*; elpd_diff for a model with no interaction = -3.3 ±3.7*se*). A non-linear smooth on temperature did not improve fit and did not exhibit pronounced nonlinearity indicated by the spline variance parameter (i.e., credible ‘wiggliness’); thus, we retained a linear fit for temperature. Finally, after performing a reloo to account for some bad pareto estimates, our final model outperformed the loo of the original model (elpd_diff = -0.2 ±0.5*se*), a model with group as a fixed effect (elpd_diff = -0.7 ±0.7*se*), and a model that excluded subject ID as a random effect (elpd_diff = -145.0 ±17.9*se*).

To select our final ‘B’ group model, we first constructed an intercept-only model, confirming the suitability of a lognormal family. Group as random effect improved fit, relative to the intercept-only model (elpd_diff = -78.5 ±15.6*se*). As with the five group model, we proceeded with the 2-day hottest maximum temperature; relative to a random effects only model, inclusion of temperature, age, and sex as fixed effects performed similarly (elpd_diff = -0.3 ±5.0*se*). The inclusion of interaction terms either performed similarly or reduced fit (relative to the elpd for a model without interaction, elpd_diff for sex*temperature = -0.7 ±0.9*se*; for a model with age*temperature = -0.9 ±0.8*se*; temperature interaction with a sex : temperature interaction = -2.4 ±0.9*se*). A non-linear smooth on temperature did not improve fit and did not exhibit pronounced nonlinearity indicated by the spline variance parameter (i.e., credible ‘wiggliness’); thus, we retained a linear fit for temperature.

S3. *Model Fit*

With the five group model, there were a small number of divergences from the posterior distribution (46 from 10,000 draws) and estimates for the intercept, as well as the group random effect intercept, had lower ESS values. Running group as a fixed effect resolved divergences and improved ESS of the intercept, though the main result remained the same – as we were not interested in group differences, we retained group as a random effect. Although 10 samples (0.8%) exhibited bad pareto k values (between 0.7 and 1.0) and 1 sample had a pareto of 1.01, *pp_check()* suggested good fit (**Supplementary Figure 3**) and Rhat values were all 1, with appropriate ESS values. High pareto values were likely driven by the subject random effect, as its removal resulted in the elimination of these high values. A model without subject as a random effect had similar outcomes to the final model with random effects. Even so, re-computing the random effect model with *reloo()* and rerunning a *loo_compare()* suggested that the final model fit better than the model without subject as a random effect.

With the 'B' group model, there were no divergences. Two samples (0.3%) exhibited bad pareto k values (between 0.7 and 1.0); even so, *pp_check()* suggested good fit (**Supplementary Figure 4**), Rhat values were all 1, and ESS values were appropriate. A model without subject as a random effect resolved bad pareto estimates, but had similar outcomes to the final model with both random effects.

For the temperature-behavior and behavior-only models, 1.2% and 0.7% of samples exhibited bad pareto k values (between 0.7 and 1.0); even so, *pp_check()* suggested good fit (**Supplementary Figures 5** & **6**), Rhat values were all 1, and ESS values were appropriate – with low tail ESS for the behavior-only model. The temperature-behavior model had 4 divergences, while the behavior-only model had 81. Models without subject as a random effect resolved bad

pareto estimates, and had similar outcomes to the final model with both random effects – though both models exhibited a positive association between age and hair cortisol without subject as a random effect. The behavior-only model without subject as a random effect had an improved tail_ESS for group.

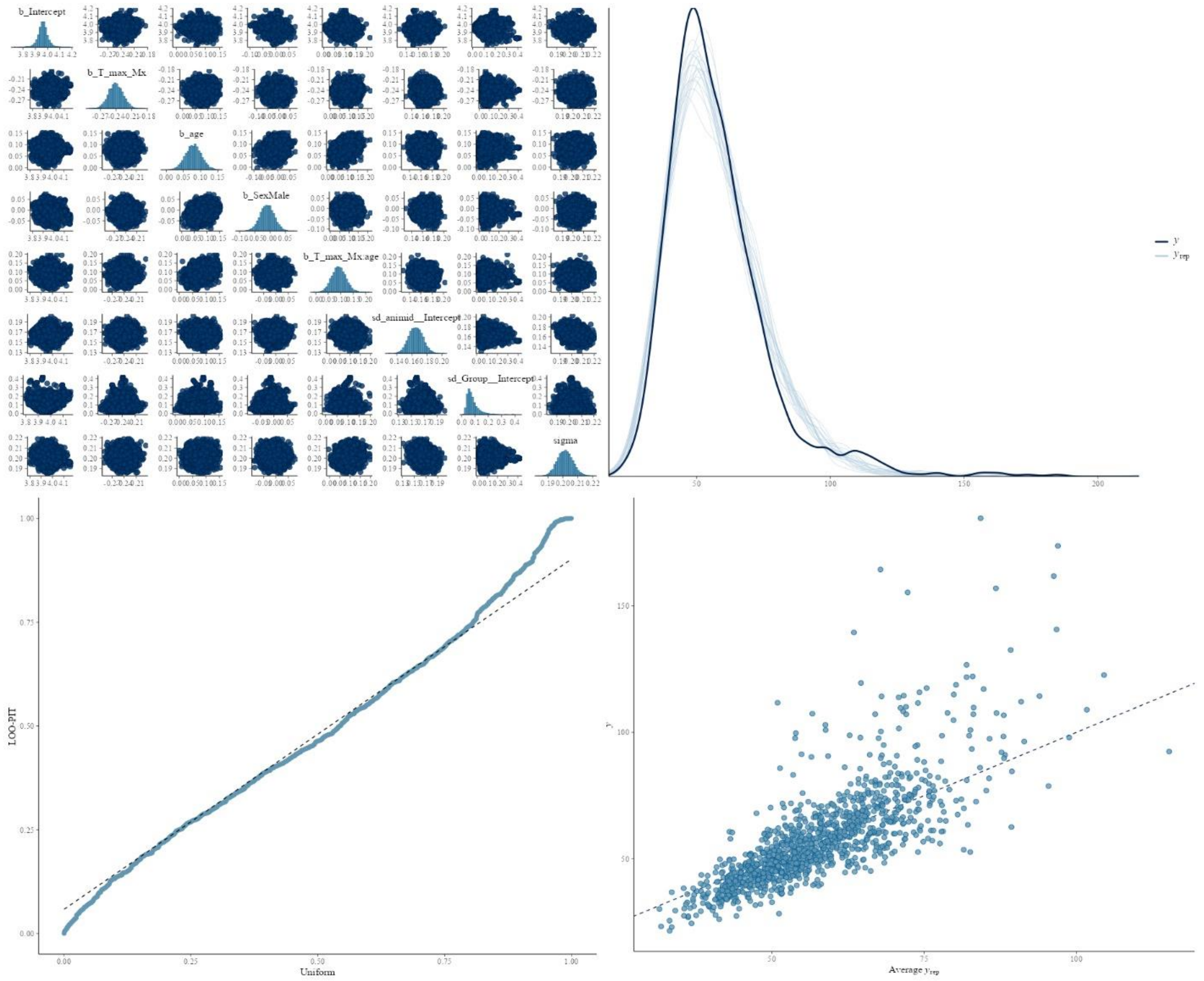

Supplementary Figure 3. Posterior predictive checks based on the full five-group model. Starting from top left and proceeding clockwise, we present our model's matrix of pairs plots, the density overlay plot, a plot of the simulated test statistics relative to the observed test statistic, and a loo pit plot (leave one out probability integral transformation).

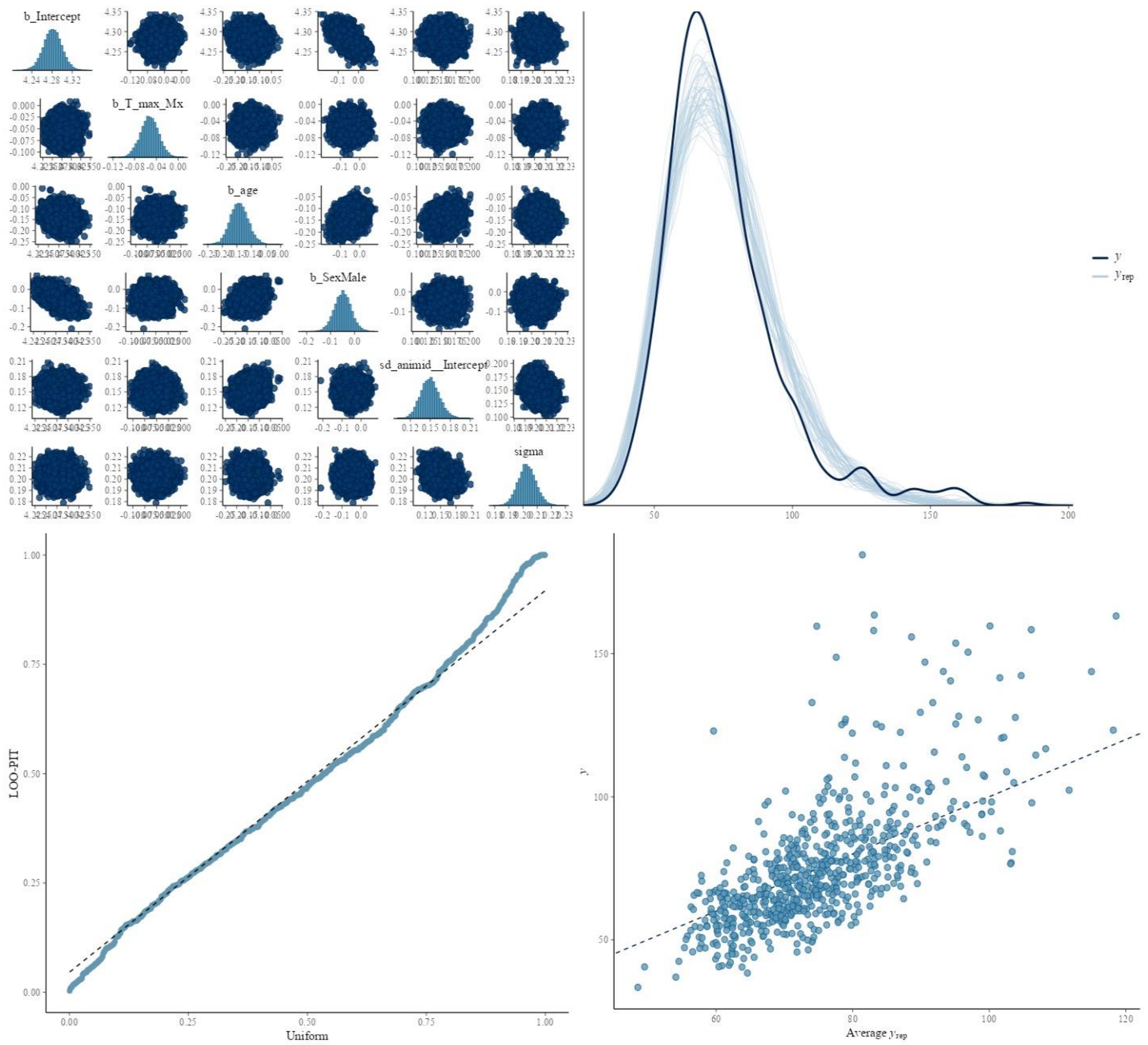

Supplementary Figure 4. Posterior predictive checks based on the full 'B' group model. Starting from top left and proceeding clockwise, we present our model's matrix of pairs plots, the density overlay plot, a plot of the simulated test statistics relative to the observed test statistic, and a loo pit plot (leave one out probability integral transformation).

S4. *Model Selection Comparisons with Behavior*

Supplementary Table 1. Model selection results for the temperature-behavior models and the behavior-only models.

*Model Identifier**	*Fixed Effects***	*elpd_diff*	*se_diff*	*Equivalent†*
	**Temperature-Behavior Models**			
B1	Agg. (Given) + Consorts + Temperature_Max + Sex + Age	0	0	-
C	Agg. (Given) + Sex + Temperature_Max + Age	-10	3.5	No
B2	Agg. (Given) + Group Agg. + Temperature_Max + Sex + Age	-11	3.8	No
B3	Agg. (Given) + Agg. (Rec.) + Temperature_Max + Sex + Age	-11.2	4.1	No
A	Agg. (Given) + Consorts + Group Agg. + Agg. (Rec.) + Temperature_Max + Sex + Age	-11.7	4.2	No
D	Sex + Temperature_Max + Age	-13.2	5.5	No
	**Behavior-Only Models**			
A	Agg. (Given) + Agg. (Rec.) + Consorts + Group Agg. + Sex + Age	0	0	-
B1	Agg. (Given) + Consorts + Sex + Age	-5.6	4.1	Yes
B2	Agg. (Given) + Group Agg. + Sex + Age	-29.7	7.4	No
B3	Agg. (Given) + Agg. (Rec.) + Sex + Age	-32.8	8.4	No
D	Sex + Age	-35.8	8.2	No
C	Agg. (Given) + Sex + Age	-35.9	8.1	No

*Models were arbitrarily identified according to complexity based on the number of fixed effects. Identifiers are consistent for the comparable behavior-only and temperature-behavior models.
**All models include random effects for group and subject identifier.
†Equivalence was set according to whether the elpd_diff exceeded a distance of 2 standard errors from the top performing model (top row). Equivalence was assessed within the temperature-behavior models and within the behavior-only models. All temperature-behavior models outperformed all the behavior-only models, with the top performing behavior-only model (A) having an elpd_diff of -147.4 ±18.3*se*, relative to the top performing temperature-behavior model (A).
Note: Aggression (given) and aggression (received) exhibited multicollinearity in the top-performing behavior-only model (A)

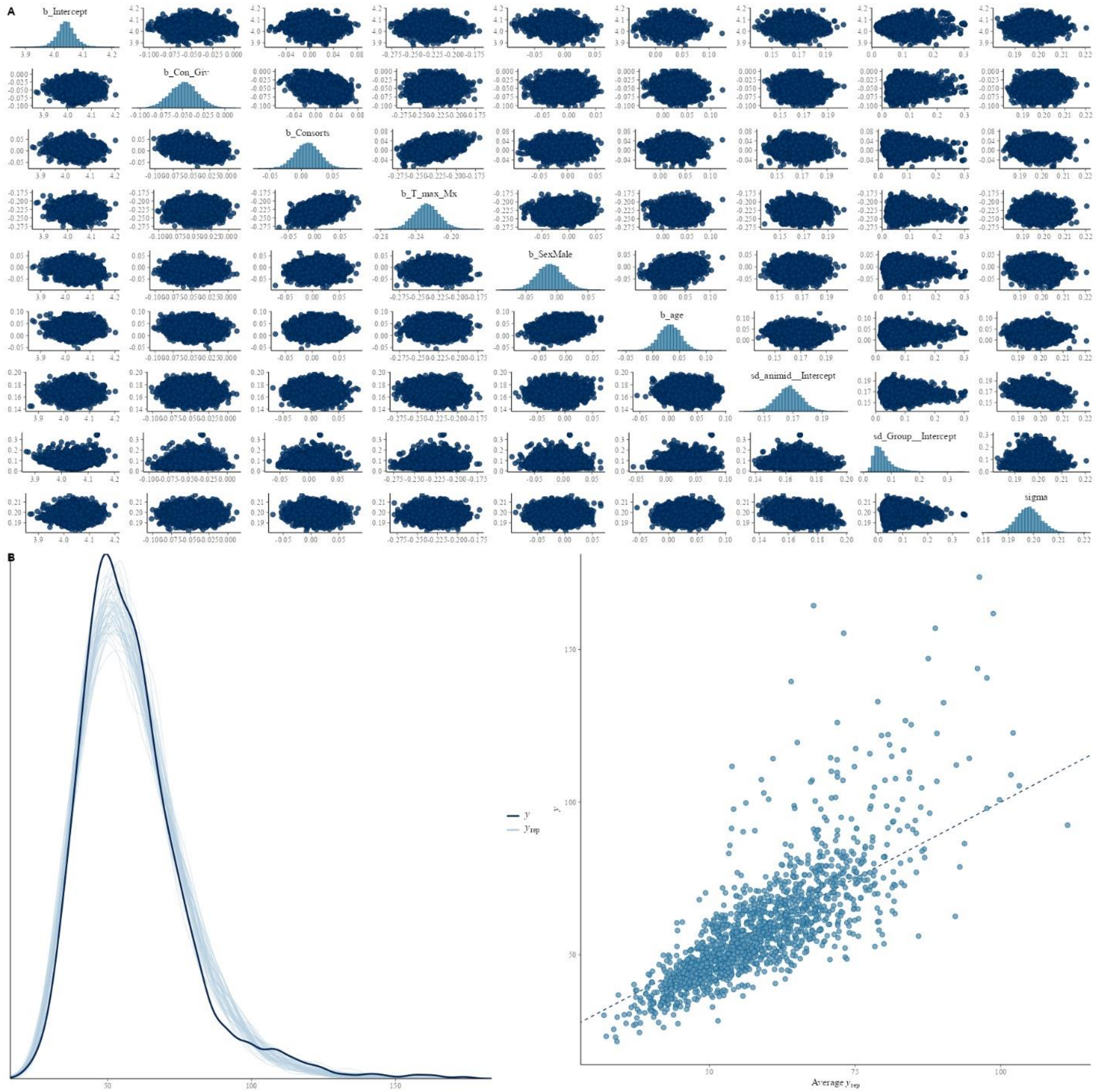

Supplementary Figure 5. Posterior predictive checks based on the full temperature-behavior model. Starting from top left and proceeding clockwise, we present our model's matrix of pairs plots, the density overlay plot, a plot of the simulated test statistics relative to the observed test statistic, and a loo pit plot (leave one out probability integral transformation).

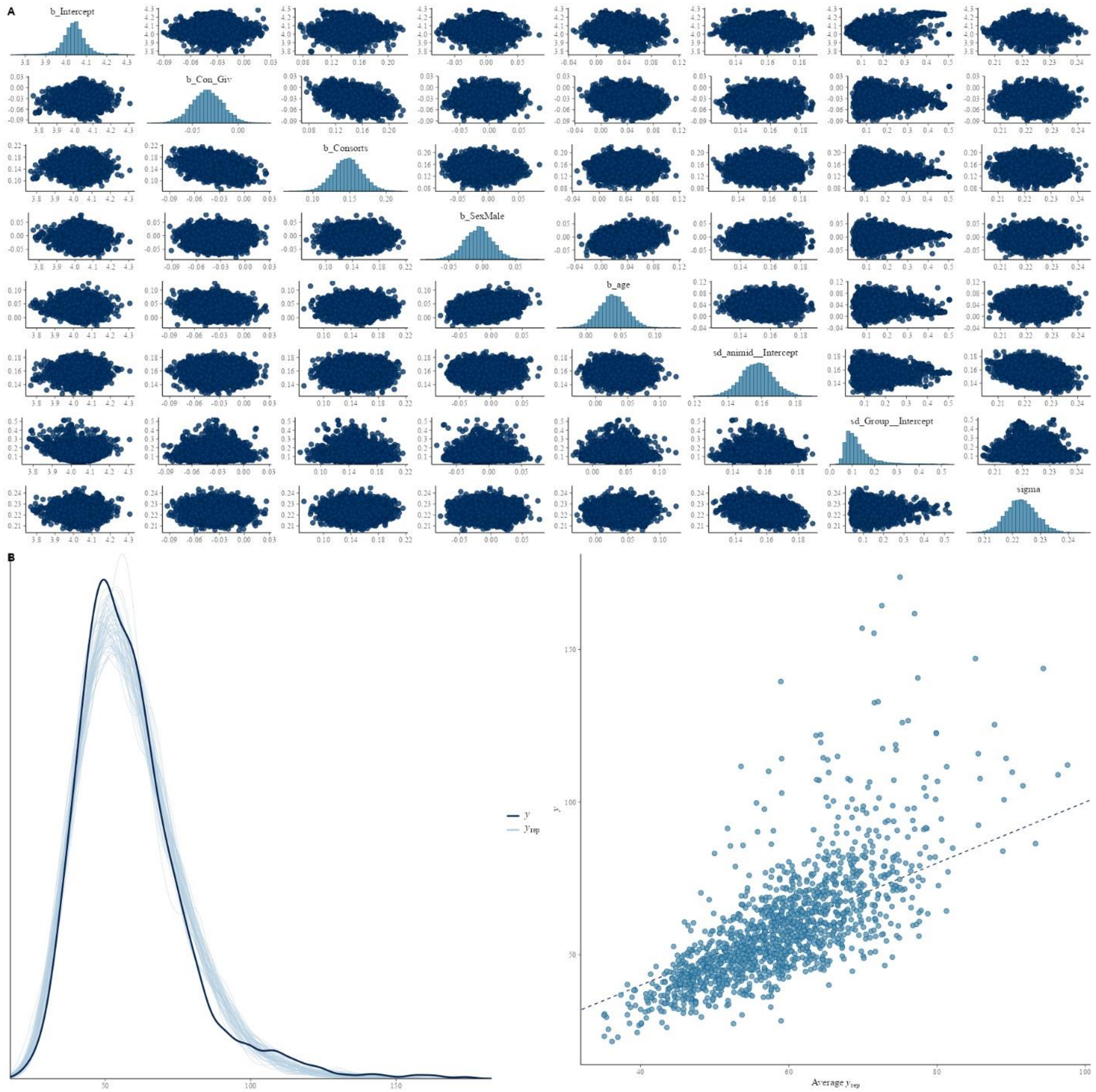

Supplementary Figure 6. Posterior predictive checks based on the full behavior-only model. Starting from top left and proceeding clockwise, we present our model's matrix of pairs plots, the density overlay plot, a plot of the simulated test statistics relative to the observed test statistic, and a loo pit plot (leave one out probability integral transformation).
